# Single-Cell Transcriptomics Reveals Evidence of Endothelial Dysfunction in the Brains of COVID-19 Patients with Implications for Glioblastoma Progression

**DOI:** 10.3390/brainsci13050762

**Published:** 2023-05-05

**Authors:** Abhimanyu Thakur, Lifan Liang, Sourav Banerjee, Kui Zhang

**Affiliations:** 1 Centre for Regenerative Medicine and Health, Hong Kong Institute of Science and Innovation—CAS Limited, Hong Kong 999077, China; 2 Department of Biomedical Informatics, University of Pittsburgh, Pittsburgh, PA 15206, USA; lil115@pitt.edu; 3 Department of Cellular and Systems Medicine, School of Medicine, University of Dundee, Dundee DD1 9SY, UK; s.y.banerjee@dundee.ac.uk; 4 State Key Laboratory of Resource Insects, College of Sericulture, Textile and Biomass Sciences, Southwest University, Chongqing 400716, China; 5 Cancer Centre, Medical Research Institute, Southwest University, Chongqing 400716, China

**Keywords:** COVID-19, SARS-CoV-2, endothelial dysfunction, brain, single-cell transcriptomic

## Abstract

Background: Endothelial dysfunction is implicated in various inflammatory diseases such as ischemic stroke, heart attack, organ failure, and COVID-19. Recent studies have shown that endothelial dysfunction in the brain is attributed to excessive inflammatory responses caused by the SARS-CoV-2 infection, leading to increased permeability of the blood-brain barrier and consequently neurological damage. Here, we aim to examine the single-cell transcriptomic landscape of endothelial dysfunction in COVID-19 and its implications for glioblastoma (GBM) progression. Methods: Single-cell transcriptome data GSE131928 and GSE159812 were obtained from the gene expression omnibus (GEO) to analyze the expression profiles of key players in innate immunity and inflammation between brain endothelial dysfunction caused by COVID-19 and GBM progression. Results: Single-cell transcriptomic analysis of the brain of COVID-19 patients revealed that endothelial cells had undergone significant transcriptomic changes, with several genes involved in immune responses and inflammation upregulated. Moreover, transcription factors were observed to modulate this inflammation, including interferon-regulated genes. Conclusions: The results indicate a significant overlap between COVID-19 and GBM in the context of endothelial dysfunction, suggesting that there may be an endothelial dysfunction link connecting severe SARS-CoV-2 infection in the brain to GBM progression.

## 1. Introduction

The novel coronavirus, SARS-CoV-2, is the causative agent of the current global pandemic of the coronavirus disease 2019 (COVID-19) [1,2]. As an epitheliotropic virus, endothelial cell invasion is a common phenomenon. Recent studies revealed that SARS-CoV-2 can invade the human brain, causing endothelial dysfunction [3]. Endothelial dysfunction is a condition characterized by an impairment of the normal functioning of endothelial cells, which line the blood vessels. When endothelial cells become damaged or dysfunctional, they are unable to produce normal amounts of nitric oxide, which is an important molecule that helps to regulate blood pressure and oxygen flow in the body. Endothelial dysfunction can also lead to an increased risk of clotting, which is associated with an increased risk of cardiovascular diseases such as hypertension, coronary artery disease, and ischemic stroke, as well as other medical conditions including diabetes and obesity. Endothelial dysfunction is caused by a variety of factors, including high blood pressure, diabetes, smoking, and inflammation. The common genes involved in endothelial dysfunction include vascular endothelial growth factor receptor-2 (*VEGFR-2*), endothelial nitric oxide synthase (*eNOS*), angiotensin converting enzyme-2 (*ACE2*), endothelin-1 (*ET-1*), endothelial protein C receptor (*EPCR*), transforming growth factor beta (*TGF-β*), angiopoietin-2 (Ang-2), tumor necrosis factor alpha (*TNF-α*), intercellular adhesion molecule-1 (*ICAM-1*), and platelet-derived growth factor (*PDGF*) [4,5,6]. VEGFR-2 is involved in the regulation of vascular permeability and the growth and migration of endothelial cells [7]. eNOS is responsible for the release of nitric oxide, which helps to regulate vascular tone and blood flow [8]. ACE2 is involved in the breakdown of angiotensin-II, a hormone that affects blood pressure [9]. ET-1 is a hormone that helps to regulate vascular tone and blood flow [8]. EPCR is involved in the regulation of inflammation and vascular permeability [10]. TGF-β is a hormone that helps to regulate cell growth, differentiation, and proliferation. Ang-2 is involved in the growth and remodeling of blood vessels [11]. TNF-α is a cytokine involved in inflammation and the regulation of immune responses [12]. ICAM-1 is involved in the regulation of inflammation and cell migration. PDGF is involved in the growth and remodeling of blood vessels [13]. Their altered expression levels are involved in endothelial dysfunction, which may contribute to the severity of COVID-19 symptoms.

The SARS-CoV-2 has been found to infect target cells, such as alveolar cells, macrophages, endothelial cells, kidney cells, intestinal epithelial cells, monocytes, neurons, glial cells, and neuroepithelial cells, when it binds to the angiotensin-converting enzyme 2 (ACE2) receptors with the aid of the S protein and transmembrane protein serine protease 2 (TMPRSS2). It is suggested that the S protein of SARS-CoV-2 contains domains that encode polybasic cleavage sites, which could account for its higher transmissibility and virulence when compared to other coronaviruses. COVID-19 is characterized by respiratory symptoms such as fever, fatigue, abnormal chest X-rays, coughing, and shortness of breath. Additionally, many patients have experienced neurological effects from the virus, including hypogeusia, dizziness, headaches, myalgia, impaired consciousness, hyposmia, seizures, and ataxia. However, the exact mechanisms by which SARS-CoV-2 invades the central nervous system (CNS) are yet to be fully understood. Recent research shows that SARS-CoV-2 may be able to enter cells in the brain via Neuropilin-1 (NRP1), CD147 (or Basigin), Cathepsin L (CTSL), and furin, which have a higher and broader expression in the brain than TMPRSS2 or ACE2. This could be one way in which SARS-CoV-2 is able to spread within the CNS. After the cellular entry, it replicates itself using its cellular machinery. SARS-CoV-2 uses ribonucleic acid (RNA) as its genetic code to replicate inside the cells of an infected host. The RNA of the virus is capped on one end, which serves several purposes, allowing the virus to create more copies of itself. The copies are then released from the host cell, where they can infect other cells. Once it has replicated enough, it can then spread to other parts of the body, including the brain. In the brain, SARS-CoV-2 targets the endothelial cells that line the blood vessels [14,15,16]. A study in non-human primates showed that SARS-CoV-2 can also invade the CNS via the olfactory route [17]. Interestingly, SARS-CoV-2’s main protease, Mpro main protease, has been found to cause microvascular brain pathology in infected individuals through the cleavage of nuclear factor-κB essential modulator (NEMO) protein in brain endothelial cells [18]. The SARS-CoV-2 virus could potentially enter the CNS through direct infection of endothelial cells and disruption of the blood-brain barrier (BBB). Data suggests that the spike protein of the virus has the potential to affect the integrity of the BBB both in vivo and in vitro [19,20]. Although it is not clear whether the free spike protein can be found in patients, these studies point to the potential of the virus to cause disturbances to the BBB through recombinant S1 proteins [21]. SARS-CoV-2 can infiltrate and damage endothelial cells, which can cause vascular endothelialitis and result in vascular thrombosis, leading to a variety of problems, such as impaired blood flow and an increased risk of ischemic stroke or dementia [22,23].

Apart from several neurological complications, the increased expression of NRP1, a cell surface receptor, in COVID-19 may lead to the progression of primary brain tumors [24]. A recent study showed that COVID-19 has been linked to an increase in neuropilin-1 expression [25]. NRP1 acts as one of the viral entry receptors beside ACE2, Ephrin (Eph), and CD147, contributing towards host cell entry of SARS-CoV-2 in the CNS and triggering intracellular signaling linked to GBM pathogenesis. Intriguingly, the Eph receptor can also contribute to GBM [26,27]. GBM is a fatal brain cancer originating from neuroglia stem cells that affect the brain and spinal cord [28,29,30]. NRP1 is involved in angiogenesis, which may explain the increased incidence of long-term neurological complications and the progression of primary brain tumors in COVID-19 patients. This may be due to the role of NRP1 in promoting tumor growth and blood vessel formation, which could worsen the prognosis of these conditions [25,31]. However, the impact of COVID-19-based endothelial dysfunction on the progression of GBM has not been examined yet.

Another important factor contributing to endothelial dysfunction in the brain is the enhanced permeability of the BBB [32]. The structure of the BBB is typically composed of astrocytes, endothelial cells, and pericytes, which play an important role in maintaining the health of the BBB [33]. A recent research study demonstrated that SARS-CoV-2 can cross the BBB through a transcellular route, which does not involve significant changes to tight junctions but can disrupt the basement membrane [34]. The increase in permeability of the BBB occurs when the tight junctions between endothelial cells are weakened, allowing increased permeability of harmful molecules and ions, such as pro-inflammatory cytokines, into the brain [35,36]. This can lead to inflammation, oxidative stress, and decreased nitric oxide production, which can reduce the ability of the endothelium to regulate blood flow, leading to numerous diseases [37]. In COVID-19, the virus is thought to affect the BBB and lead to endothelial dysfunction through the secretion of pro-inflammatory cytokines and chemokines, which can lead to disruption of the BBB and cause inflammation of the endothelium, which can lead to endothelial dysfunction. Major genes regulating the permeability of the BBB include *CLDN5* [38], *AQP4* [39,40], *F11R* [41], and *SCUBE2* [42,43]. Apparently, their cell type-specific distribution and expression level analysis are crucial in the brain tissue of COVID-19 patients.

Single-cell transcriptomics has become an essential technique for identifying the link between COVID-19-based endothelial dysfunction and its impact on GBM progression. It enabled us to analyze the genetic and molecular profiles of individual cells as well as gain insight into their interactions with other cells and the environment. By leveraging single-cell transcriptomic data, we can identify the genes and pathways associated with endothelial dysfunction and GBM progression and understand how these two conditions are linked. Furthermore, this data can help us develop targeted treatments for both conditions. Ultimately, single-cell transcriptomics is an invaluable tool for uncovering the molecular basis of COVID-19-based endothelial dysfunction and its impact on GBM progression and should be leveraged to its full potential.

Recently, single-cell transcriptomic studies have gained tremendous attention in biomedical research as they allow a better understanding of cell-to-cell variability, gene expression networks, and gene regulatory dynamics and can provide insights into diseases at the single-cell level [44]. Additionally, it can help in the identification of novel biomarkers, drug targets, and cell types. It has enabled the analysis of the genetic and molecular profiles of individual cells as well as insight into their interactions with other cells and the environment. Thus, single-cell transcriptomic studies are vital for advancing our understanding of biology and improving the diagnosis and treatment of diseases [45,46]. Therefore, by leveraging the value of single-cell transcriptomic data, we aim to identify the genes and pathways associated with endothelial dysfunction in COVID-19 and its impact on GBM progression and understand how these two conditions are linked. This will potentially unfold the new avenue of connection between COVID-19 and GBM via endothelial dysfunction as a common inflammatory phenomenon.

## 2. Materials and Methods

### 2.1. Single Cell RNA-Seq Dataset

Single-cell RNA sequencing datasets GSE131928 and GSE159812 were obtained from the Gene Expression Omnibus (GEO), a public database accessible at https://www.ncbi.nlm.nih.gov/geo/ (accessed on 10 February 2023). The GSE131928 dataset features data from 28 GBM patients, while GSE159812 consists of samples from 30 patients diagnosed with COVID-19, influenza, or non-viral conditions. The analysis focused on 13 non-viral control patients and 15 COVID-19 patient samples from the GSE159812 dataset.

### 2.2. Computational Analysis, Statistics, and Schematics

The single-cell RNA-seq datasets were analyzed with the statistical programming language R (v. 4.4.2). The following packages are used for loading, saving, and manipulating data, as well as data integration, analysis, and generating plots: Seurat (v 4.3.0), tidyverse (v 1.3.2), dplyr (v 1.1.0), patchwork (v 1.1.2), stringr (v 1.5.0), Harmony (v 0.1.1), org.Hs.eg.db (v 3.16.0), and SingleR (v 2.0.0). Briefly, the raw data of the single cell datasets (GSE159812 and GSE131928) were downloaded from the GEO (https://www.ncbi.nlm.nih.gov/geo/ (accessed on 30 March 2023)). The read count matrix was read into R, which subsequently created the Seurat object with the CreateSeuratObject () function. According to the number of genes, the percentage of mitochondrial genes, and the percentage of erythrocyte genes in the samples. The cells with >300 genes, a mitochondrial gene percentage of <10%, and an erythrocyte gene percentage of <3% were reserved and used for the following analysis: To remove the batch effect, all samples were integrated iteratively. Uniform manifold approximation projections (UMAPs) or t-distributed stochastic neighbor embedding (t-SNE) were then calculated using the runUMAP() or RunTSNE() functions, respectively, using the first 20 dimensions. The analysis results were visualized using the FeaturePlot(), VlnPlot(), and DotPlot() functions. The single cell portal (available at https://singlecell.broadinstitute.org/ (accessed on 30 March 2023)) was also utilized for generating tSNE maps and violin plots.

Differentially expressed genes (DEGs) were derived from COVID-19 scRNA-seq (GSE164485). We performed differential gene expression analysis for each cell type labeled by the original study, comparing patient samples with control samples. The testing method was the Wilcoxon rank sum test. Benjamin-Hochberg was used for multiple testing corrections. The adjusted *p*-value cut-off was 0.05. Then we selected the top 100 genes correlated with the fetal brain road map’s three diffusion components (DC) aligned to the GBM cells. Further, we performed enrichment analysis to investigate how cell type-specific DEGs in COVID-19 overlap with genes that participate in GBM development. Enrichment analysis was conducted using the function “enriched ()” in the R package “clusterProfiler”.

## 3. Results

### 3.1. Annotation of Major Brain Cell Types from Healthy and COVID-19 Patients’ Brain

**Figure 1:**
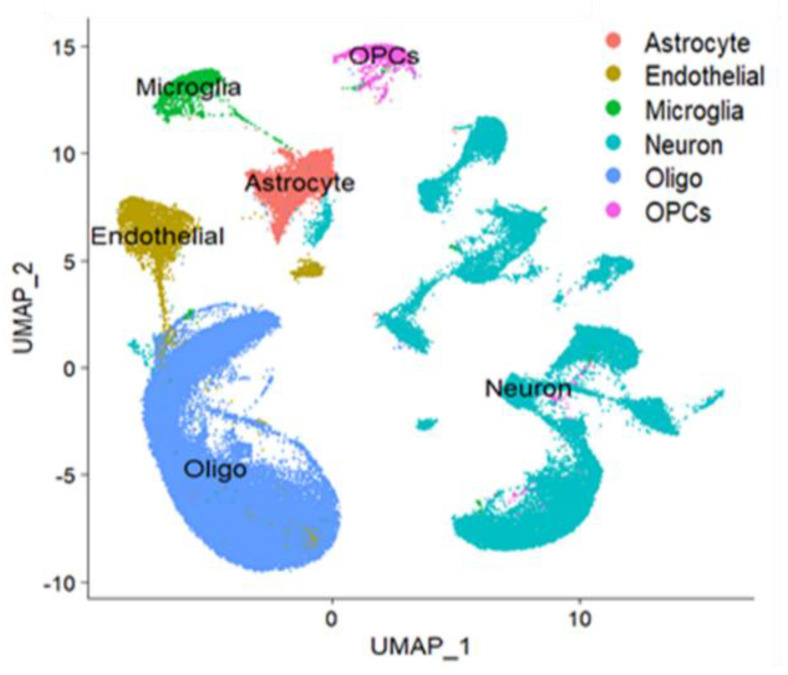
UMAP based clustering of brain cells from the brain samples of healthy and COVID-19 patients. Representative UMAP showing the cluster of major brain cell types, namely astrocytes, endothelial cells, microglia, neurons, oligodendrocytes (Oligo), and oligodendrocyte progenitor cells (OPCs), clustered based on their marker.

Endothelial dysfunction is a condition in which the inner lining of the blood vessels is not functioning properly, which can lead to a variety of health issues, including an increased risk of clotting, heart attack, ischemic stroke, and other cardiovascular complications. Endothelial dysfunction is believed to be one of the major contributing factors to the severity of COVID-19, as it can impair the immune system’s ability to fight the virus, leading to more severe symptoms [47,48]. Single-cell data analysis can help identify the underlying mechanisms of endothelial dysfunction in COVID-19 patients as well as its severity. By examining individual cells from the brain, researchers can determine the presence of any abnormal biological pathways or molecules that could be contributing to the condition. Additionally, they can also look at how the cells are responding to the virus and how this response is impacting the severity of the patient’s symptoms [49]. Apparently, single-cell data analysis of healthy and COVID-19 patients’ brains is necessary to better understand the underlying transcriptomic landscape of endothelial dysfunction in COVID-19 and its severity. Therefore, to gain a thorough comprehension of the impacts of COVID-19 on the brain, we implemented single-cell RNA sequencing (scRNA-seq) to create an atlas of the brain. This atlas identified 43,231 and 54,988 nuclei from the brains of healthy individuals and COVID-19 sufferers, respectively. We employed a three-part methodology for cell-type identification. First, we conducted an unbiased search for cluster markers. Second, we used signatures from published atlases to identify cell types. Finally, we manually sub-stratified cell populations and cell states using expert knowledge [50,51]. To gain further insight, we applied principal component analysis (PCA) to generate UMAP representations of the major brain cell types, including astrocytes (*n* = 2586), endothelial cells (*n* = 960), microglia (*n* = 1016), neurons (*n* = 11,853), oligodendrocytes (*n* = 25,880), and oligodendrocyte progenitor cells (OPCs) (*n* = 936) (Figure 1). Subsequently, the annotated clusters were utilized to examine the cell-type-specific distribution of genes contributing to endothelial dysfunction.

### 3.2. Aberrant Expression of CLDN5 in Endothelial Cells during COVID-19

**Figure 2:**
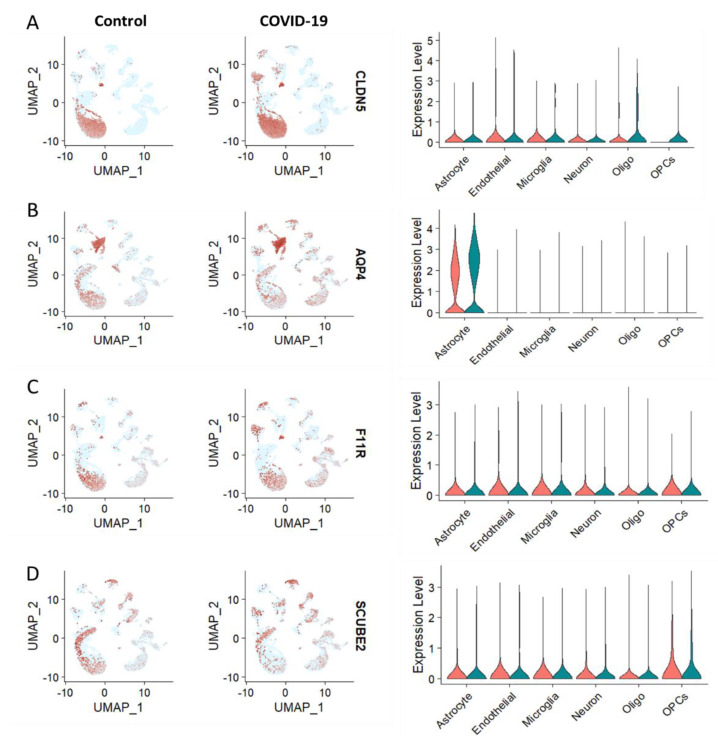
CLDN5 is suppressed in endothelial cells under COVID-19 infection. Representative UMAPs and violin plots showing the cell type-specific distribution and expression level of (**A**) CLDN5, (**B**) AQP4, (**C**) F11R, and (**D**) SCUBE2 in the brain of COVID-19-infected patients as compared to the healthy control. In violin plots, pink and green color represents control and COVID-19 groups, respectively.

The BBB is an important protective layer that helps protect the brain from potentially dangerous substances circulating in the bloodstream. However, recent studies have suggested that COVID-19 infections may be associated with BBB leakage. This could potentially lead to a range of neurological complications, including inflammation, brain swelling, and seizures [52]. Several proteins, including claudin-5, occludin, junctional adhesion molecule-A (JAM-A) (or F11R), aquaporin-4, and cerebral endothelial glycoprotein-1 (CEGP-1), have been found to play crucial roles in maintaining the structural integrity and semi-permeability of the BBB [38,39,40,41,42,43]. Therefore, we intend to examine their cell type-specific distribution and expression level in the major brain cells of COVID-19 patients. Importantly, the expression level of CLDN5, which encodes Claudin-5, has been found to be decreased in endothelial cells under COVID-19 infection conditions as compared to the control group, as evident from the UMAP and violin plots (Figure 2A). From Figure 2B–D, the cell type-specific distribution of AQP4, F11R, and SCUBE2 is apparent from the respective UMAP; however, there is no significant change under the COVID-19 condition as compared to that in the control group. The altered expression of CLDN5 in endothelial cells can be attributed to the COVID-19 infection. As CLDN5 plays a vital role in the formation of the BBB by maintaining tight junctions between astrocyte and endothelial cells and is also responsible for the regulation of the permeability of this barrier, which serves to protect the brain from potentially harmful foreign substances that can enter the bloodstream, the aberrant expression of CLDN5 can potentially lead to the leakage of the BBB, which may pave the way for the entry of SARS-CoV-2 inside the brain, which is also supported by others’ studies [34,38,53].

### 3.3. Brain Cell Type-Specific Altered Expression of Major Genes Involved in Endothelial Dysfunction in COVID-19

**Figure 3:**
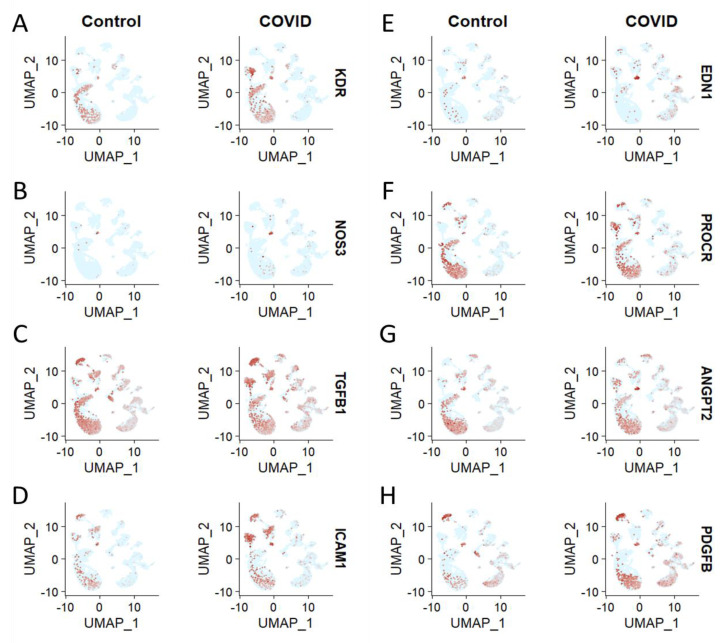
Brain cell type specific distribution of major genes, contributing to the endothelial dysfunction in COVID-19. Representative UMAPs showing the cell type-specific expression of (**A**) KDR, (**B**) NOS3, (**C**) TGFB1, (**D**) ICAM1, (**E**) EDN1, (**F**) PROCR, (**G**) ANGPT2, and (**H**) PDGFB in the brains of healthy control and COVID-19 patients.

As noted previously, KDR, NOSS3, TGFB1, ICAM1, EDN1, PROCR, ANGPT2, and PDGFB are involved in the endothelial dysfunction in COVID-19 [4,5,6]. However, their distribution in different brain cells needs to be investigated to understand their potential role in endothelial cell dysfunction after the SARS-CoV-2 infection. The distribution analysis showed that KDR, NOS, TGFB1, ICAM1, EDN1, PROCR, ANGPT2, and PDGFB are upregulated in endothelial cells of COVID-19 patients’ brains compared to the healthy control (Figure 3A–H). KDR (or VEGFR-2) is a receptor protein involved in the regulation of endothelial cell growth and survival. It is expressed in endothelial cells and is essential for angiogenesis, the formation of new blood vessels. In response to SARS-CoV-2 infection, it may be upregulated to facilitate the growth of new blood vessels, which can lead to endothelial dysfunction [54,55]. NOSS3 is an enzyme involved in the production of nitric oxide, which is a molecule that plays an important role in regulating vascular tone. In response to SARS-CoV-2 infection, NOSS3 may be downregulated, leading to decreased production of nitric oxide and subsequent endothelial dysfunction [37]. Transforming growth factor beta-1 (TGFB1) is a cytokine involved in the regulation of inflammation and the growth and differentiation of cells. It is expressed in endothelial cells and is important for the proper functioning of the vascular system. In response to SARS-CoV-2 infection, TGFB1 may be upregulated, leading to excessive inflammation and endothelial dysfunction [56]. ICAM1 is an adhesion molecule expressed on the surface of endothelial cells. It plays an important role in the regulation of leukocyte adhesion to the endothelium and, thus, in the inflammatory response. In response to SARS-CoV-2 infection, ICAM1 may be upregulated, leading to increased leukocyte-endothelial cell adhesion and subsequent endothelial dysfunction [57]. EDN1 is a peptide hormone involved in the regulation of vascular tone and inflammation. In response to SARS-CoV-2 infection, EDN1 may be upregulated, leading to increased vascular tone and inflammation and subsequent endothelial dysfunction [58]. PROCR is a membrane protein expressed on the surface of endothelial cells and plays an important role in the regulation of the coagulation cascade. In response to SARS-CoV-2 infection, PROCR may be upregulated, leading to increased activation of the coagulation cascade and subsequent endothelial dysfunction [59,60]. Angiopoietin-2 (ANGPT2) is a peptide hormone involved in the regulation of vascular permeability. In response to SARS-CoV-2 infection, ANGPT2 may be upregulated, leading to increased vascular permeability and subsequent endothelial dysfunction [6,61]. Platelet-derived growth factor B (PDGFB) is a growth factor involved in the regulation of angiogenesis and the growth and differentiation of cells. In response to SARS-CoV-2 infection, PDGFB may be upregulated, leading to increased angiogenesis and endothelial dysfunction [62]. However, NOS and PDGFB are upregulated in the oligodendrocytes of COVID-19 patients’ brains compared to the healthy control (Figure 3B,H). Additionally, EDN1 was found to be upregulated in the neurons of COVID-19 patients’ brains compared to the healthy control (Figure 3E).

### 3.4. Enhanced Expression of Major Genes Involved in Endothelial Dysfunction in ACE2 Positive Brain Cells of COVID-19 Patients

**Figure 4:**
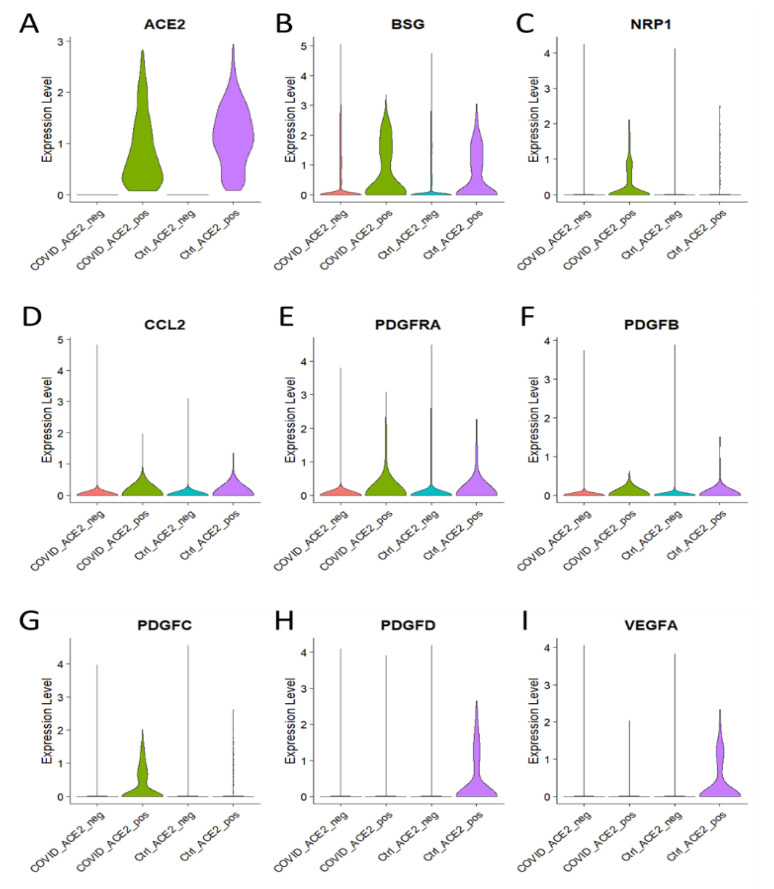
Genes involved in endothelial dysfunction are increased in ACE2 positive brain cells of COVID-19 patients. (**A**–**I**) Representative violin plots showing the expression level of (**A**) ACE2, (**B**) BSG, (**C**) NRP1, (**D**) CCL2, (**E**) PDGFRA, (**F**) PDGFB, (**G**) PDGFC, (**H**) PDGFD, (**I**) VEGFA in ACE2− or ACE2+ brain cells of healthy control and COVID-19 patients.

Endothelial dysfunction is a key factor in the development of cardiovascular and cerebrovascular diseases. Recent studies have shown that ACE2, the receptor for SARS-CoV-2, is expressed by brain microvascular endothelial cells. This suggests that ACE2 may be involved in endothelial dysfunction in COVID-19 patients [14,15,23]. To better understand the role of ACE2 in endothelial dysfunction in COVID-19 patients, it is pertinent to investigate the expression of major genes, including ACE2, AT1R, and ACE, that are involved in endothelial dysfunction in ACE2-positive brain cells. In a recent study, researchers used a mouse model to examine the role of ACE2 in endothelial dysfunction in the brain. They found that ACE2 expression was significantly increased in ACE2-positive brain cells in COVID-19 patients compared to non-COVID-19 patients. Moreover, the expression of AT1R and ACE was also significantly increased in ACE2-positive brain cells of COVID-19 patients. This suggests that ACE2 may be involved in the development of endothelial dysfunction in COVID-19 patients [23,63]. Evidently, the violin plot-based analysis and the heatmap analysis showed significantly higher expression of BSG and NRP1 in ACE2-positive COVID-19-infected brain cells as compared to the control group (Figure 4A–C). Interestingly, BSG as well as NRP1 have been reported to facilitate the host cell entry of SARS-CoV-2 [64,65]. Further, the expression of genes involved in endothelial dysfunction, namely CCL2, PDGFRA, PDGFB, and PDGFC, is found to be increased in ACE2-positive brain cells of COVID-19 patients (Figure 4D–I). Apparently, the upregulation of genes related to endothelial dysfunction in ACE2-positive brain cells suggests the involvement of ACE2-positive cells in endothelial dysfunction in COVID-19.

### 3.5. C1qRs and MAC Are Overexpressed in KEGG Pathway in COVID-19

**Figure 5:**
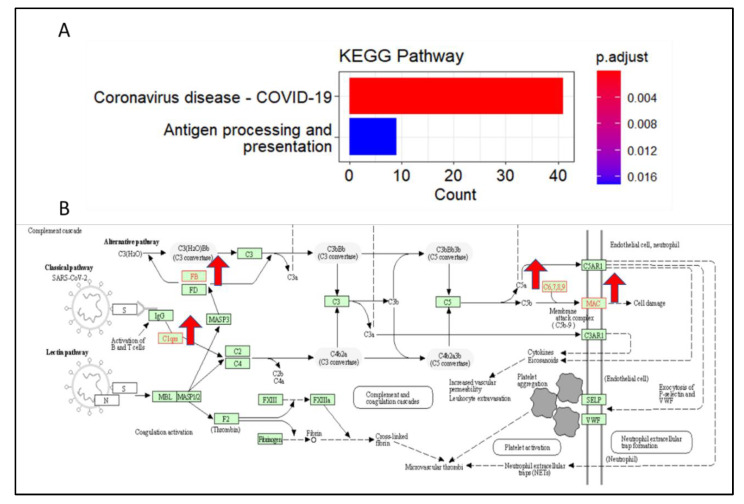
KEGG analysis for endothelial cell cluster between healthy and COVID-19 patients (**A**) Representative histogram displaying the differentially expressed genes (DEGs) via KEGG analysis. (**B**) Image of the KEGG signaling pathway according to the imported list of genes differentially expressed in the brain of COVID-19 as compared to healthy control. Red up arrow represents up-regulated genes.

KEGG pathway analysis is a bioinformatics tool that allows users to analyze gene and protein functions within a biological pathway. It allows users to search and visualize gene and protein functions within pathways, to identify their roles in a particular pathway, and to compare gene and protein functions between pathways. KEGG pathway analysis is a powerful tool for understanding the molecular mechanisms of biological processes and diseases and for drug discovery and development [66,67]. KEGG pathway analysis facilitates the understanding of high-level functions and utilities of the biological system, such as the cell, the organism, and the ecosystem, from molecular-level information, especially large-scale molecular datasets generated by genome sequencing and other high-throughput experimental technologies [68,69]. Therefore, to better understand which biological pathway the identified gene signature holistically drives, we utilized the KEGG analysis. Importantly, the KEGG pathway analysis demonstrated the involvement of COVID-19-associated genes in the majority of genes (Figure 5A). Furthermore, FB, C1qrs, and membrane attack complex (MAC) are found to be upregulated in COVID-19 (Figure 5B). Interestingly, C1q binding to C1qrd has been shown to stimulate endothelial cells to express adhesion molecules and to release chemokines and IL-6 [70]. Endothelial cell activation by the MAC has relevance to complement-dependent inflammatory processes [71].

### 3.6. Brain Cell Type-Specific Altered Expression of CLDN5, c1qRs, and C5 in GBM Patient

**Figure 6:**
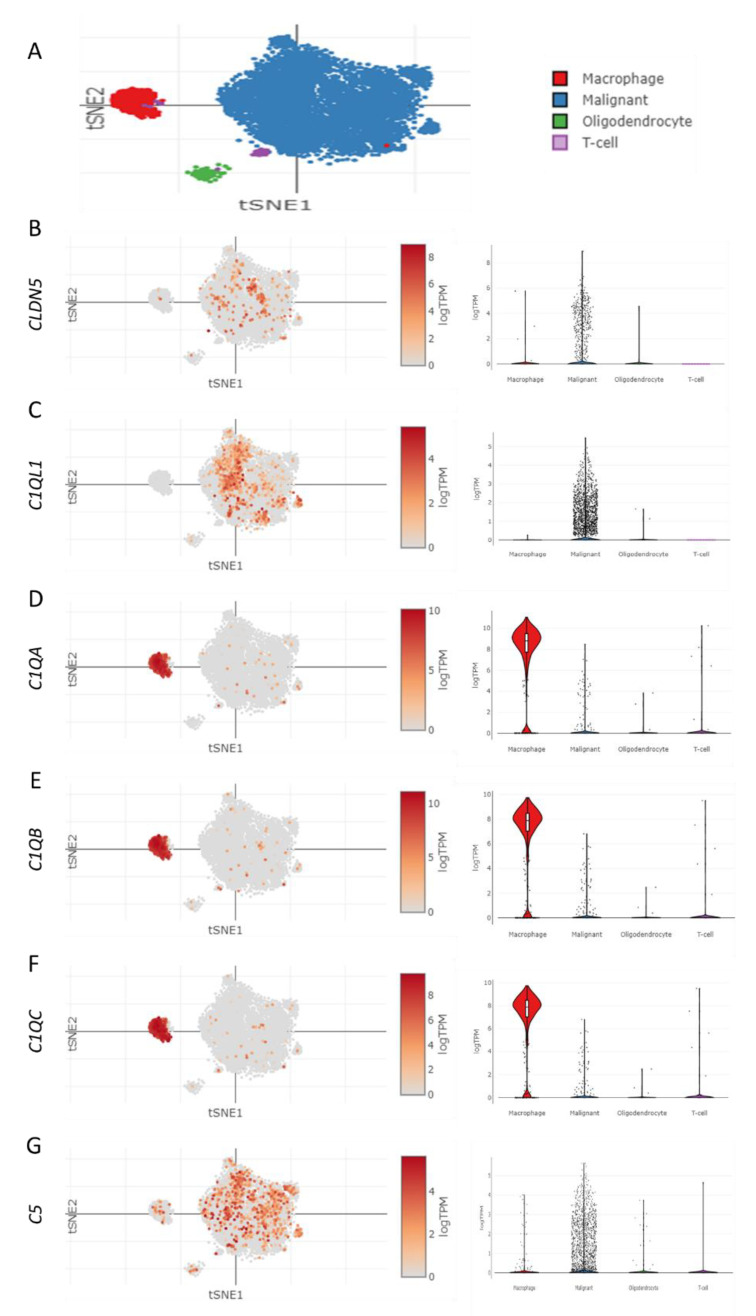
Single cell transcriptomic analysis showed altered expression of CLDN5, c1qRs, and C5 in the brains of GBM patients. (**A**) Representative tSNE map showing the cluster of major brain cell types, namely macrophages, malignant GBM cells, oligodendrocytes, and T-cells, clustered based on their marker. (**B**–**G**) Representative tSNE maps and violin plots showing the distribution and expression level of (**B**) CLDN5, (**C**) C1QL1, (**D**) C1QA, (**E**) C1QB, (**F**) C1QC, and (**G**) C5.

As noted previously, the expression levels of CLDN5, c1qRs, and C5 have been found to be altered in endothelial cells in COVID-19 patients, which has also been reported to play a role in endothelial dysfunction. Therefore, it is imperative to examine their distribution and expression level at the single cell level in the brain of GBM patients by utilizing the scRNA-seq dataset of COVID-19 patients’ brains [72]. Figure 6A shows the tSNE map depicting the clusters of different brain cells, including macrophages, malignant GBM cells, oligodendrocytes, and T-cells. The tSNE map-based distribution analysis and violin plot showed the prevalent distribution and significant expression level of CLDN5 in malignant GBM cells (Figure 6B). Our results showed alignment with the existing literature, as CLDN5 has been identified as a marker for GBM prognosis and is overexpressed in tumor cells compared to normal tissue. It is believed to be involved in the regulation of cell adhesion, migration, and invasion, which are essential for GBM progression. Therefore, it is believed that CLDN5 may be an important target for the development of novel therapeutic strategies for GBM [38,73,74,75]. Further, the distribution pattern and expression level of various c1qRs were examined, such as C1QL1, C1QA, C1QB, and C1QC. Interestingly, the level of C1QL1 was found to be prominently expressed in malignant GBM cells compared to other brain cell types (Figure 6C). Importantly, C1QL1 has been shown to be an early diagnostic marker for GBM [76], suggesting its role in GBM progression. On the contrary, C1QA, C1QB, and C1QC were majorly expressed in malignant GBM cells as well as macrophages, although significantly more in macrophages (Figure 6D–F). In the same vein, existing literature suggests that the potential of C1q produced and released by microglia/macrophage cells can promote immunosuppression, favoring the proliferation of GBM cells [77]. We further found that the level of C5 was significantly enhanced in malignant GBM cells compared to other brain cell types (Figure 6G). This conforms with the evidence that C5a is produced in large amounts by GBM microenvironments, particularly mesenchymal stem-like cells. It binds to its receptor, C5aR1, and stimulates a range of aggressive behaviors, including invasion and migration of GBM cells and GBM stem cells (GSCs) [78]. The complement system plays a key role in innate immunity, contributing to the progression of GBM. GSCs in GBM tumors can self-renew and cause tumor growth. Evidence suggests the complement system impacts GSC niches and helps to maintain them. Thus, the role of the complement system in glial tumors, particularly GSCs, is important [79].

## 4. Discussion

COVID-19 has been linked to an increased risk of endothelial dysfunction. Endothelial dysfunction is an impairment of the cells that line the inner surface of blood vessels, which in turn can lead to an increased risk of cancer [25]. It is thought that endothelial dysfunction caused by COVID-19 can cause an imbalance in the body’s immune system, leading to inflammation and abnormal cell growth. This can increase the risk of cancer as well as other cardiovascular diseases [80]. Additionally, there is evidence that individuals who have tested positive for COVID-19 have an increased risk of developing certain types of cancer. Therefore, it is important to be aware of the potential risks associated with COVID-19 and endothelial dysfunction and to take the necessary steps to reduce the risk of cancer [81]. Intriguingly, our single-cell transcriptomics analysis showed the aberrant expression of *CLDN5* in endothelial cells during COVID-19. However, the expression level of CLDN5 was found to be decreased in endothelial cells under COVID-19 infection conditions when compared with the control group, as shown by the UMAP and violin plots (Figure 2A). The UMAPs of *AQP4*, *F11R*, and *SCUBE2* demonstrate the cell type-specific distribution of these proteins, but with no significant change under the COVID-19 condition when compared to the control group (Figure 2B–D). Apparently, it can be hypothesized that the altered expression of CLDN5 in endothelial cells is a result of the COVID-19 infection. Brain cell type-specific altered expression of major genes involved in endothelial dysfunction in COVID-19. The distribution analysis showed that *KDR*, *NOS*, *TGFB1*, *ICAM1*, *EDN1*, *PROCR*, *ANGPT2*, and *PDGFB* are upregulated in endothelial cells of COVID-19 patients’ brains compared to the healthy control (Figure 3A–H). Further, the analysis of ACE2-positive brain cells from COVID-19 patients showed that the expression of major genes involved in endothelial dysfunction was significantly higher than in control cells. Through a violin plot-based analysis and a heatmap analysis, it was revealed that the genes BSG and NRP1 were both highly expressed in ACE2-positive cells infected with SARS-CoV-2. It is noteworthy that BSG and NRP1 have both been reported to facilitate the entry of SARS-CoV-2 into host cells [64,65]. Additionally, the expression of genes associated with endothelial dysfunction—namely CCL2, PDGFRA, PDGFB, and PDGFC—was also found to be increased in ACE2-positive brain cells of COVID-19 patients. These findings suggest that ACE2-positive cells are likely involved in endothelial dysfunction in COVID-19 patients (Figure 4A–D). The KEGG pathway analysis of COVID-19 has revealed that *FB*, *C1qrs*, and membrane attack complex (MAC) are significantly overexpressed in comparison to other pathways. This indicates that these three genes are likely to be involved in the pathogenesis of the virus. Further, it was observed that most of the COVID-19-associated genes were found to be upregulated. This suggests that these genes may be playing a significant role in the disease’s development. Interestingly, it was found that C1qrs and MAC were among the most significantly overexpressed genes in the KEGG pathway analysis. This provides an important insight into the molecular pathways involved in the development of COVID-19 and could potentially provide useful information for the development of therapeutic strategies (Figure 5A,B).

COVID-19 has been associated with an increased risk of GBM. This risk is linked to virus-induced endothelial dysfunction, which is the impairment of the blood vessels that transport blood to the brain [25,27,31,82] Endothelial dysfunction leads to an impaired BBB, which allows the virus to cross into the brain and cause inflammation and damage. This damage can cause mutations in the cells that can lead to GBM. Several studies have been conducted to understand the impact of COVID-19-based endothelial dysfunction on GBM. These studies have revealed that COVID-19 infection can lead to an increased risk of GBM due to virus-induced endothelial dysfunction. The virus can damage the endothelium, leading to an impaired blood-brain barrier. This allows the virus to cross the barrier and cause inflammation and damage in the brain, which can cause mutations in the cells and lead to GBM [16,83]. Notably, the single-cell transcriptomics study of GBM patients revealed altered expression of CLDN5, c1qRs, and C5 in brain cell types. To further analyze the expression patterns of these molecules, a tSNE map was made depicting the clusters of different brain cells, including macrophages, malignant GBM cells, oligodendrocytes, and T-cells (Figure 6A). The subsequent distribution analysis and violin plot (Figure 6B) revealed *CLDN5* to be predominantly expressed in malignant GBM cells. The expression levels of four *C1qRs-C1QL1*, *C1QA*, *C1QB*, and *C1QC*- were also examined. It was observed that *C1QL1* was prominently expressed in malignant GBM cells in comparison to other cell types (Figure 6C). *C1QL1* has been previously identified as an early diagnostic marker for GBM, suggesting its involvement in GBM progression. On the other hand, while *C1QA*, *C1QB*, and *C1QC* were expressed in both malignant GBM cells and macrophages, their expression levels were significantly higher in the latter (Figure 6D–F). A key oncogenic driver of GBM is the receptor tyrosine kinase PDGFRA, which binds to its ligands PDGFA/B/C to trigger growth factor signaling and inhibition of mitophagy in oligodendroglial progenitor cell-like GBM stem cells, resulting in pro-neural GBM [72,84,85]. Clinical trials for pediatric solid tumors, including CNS tumors, are underway with drugs targeting PDGFRA (NCT04773782). The upregulation of PDGFRA and its ligands in COVID-19 suggests that this pathway could be a key therapeutic target for both COVID-19 and GBM. Evidently, this study found that the expression profiles of significant molecules associated with innate immunity and inflammation are in correspondence with both GBM progression and the brain endothelial dysfunction caused by SARS-CoV-2 infection. This implies that severe SARS-CoV-2 infection in the brain may be a potential factor in predisposing people to GBM progression. Therefore, this study suggests that the link between endothelial dysfunction and GBM progression should be further investigated. Although the results obtained via scRNA-seq analysis were promising, they warrant further research with a large number of datasets given the limited sample size of 28 GBM samples, 13 of which were non-viral and 15 of which were COVID-related.

## 5. Conclusions

COVID-19 has been linked to an increased risk of endothelial dysfunction, an impairment of the cells that line the inner surface of blood vessels, which can lead to an increased risk of GBM. Single-cell transcriptomics analysis has revealed the aberrant expression of CLDN5 in endothelial cells during COVID-19, with the expression level of CLDN5 decreasing in endothelial cells compared to the control group. Additionally, brain cell type-specific altered expression of major genes involved in endothelial dysfunction in COVID-19 was observed, with KDR, NOS, TGFB1, ICAM1, EDN1, PROCR, ANGPT2, and PDGFB upregulated in endothelial cells of COVID-19 patients compared to the healthy control. ACE2-positive brain cells from COVID-19 patients showed that expression of major genes involved in endothelial dysfunction was also significantly higher than in control cells, with BSG and NRP1 highly expressed in ACE2-positive cells infected with SARS-CoV-2. Moreover, the KEGG pathway analysis revealed that FB, C1qrs, and the membrane attack complex (MAC) are significantly overexpressed in comparison to other pathways. These findings suggest that COVID-19 infection can lead to an increased risk of GBM due to virus-induced endothelial dysfunction. It is believed that the virus can damage the endothelium, leading to an impaired blood-brain barrier, which allows the virus to cross into the brain and cause inflammation and damage. A single-cell transcriptomics study of GBM patients revealed altered expression of CLDN5, c1qRs, and C5 in brain cells, with CLDN5 predominantly expressed in malignant GBM cells and C1QL1 an early diagnostic marker for GBM. These findings imply that severe SARS-CoV-2 infection in the brain may be a potential factor in predisposing people to GBM progression and suggest that the link between endothelial dysfunction and GBM progression should be further investigated.

## Data Availability

Single-cell RNA sequencing datasets GSE131928 and GSE159812 were obtained from the Gene Expression Omnibus (GEO), a public database accessible at https://www.ncbi.nlm.nih.gov/geo/ (accessed on 30 March 2023).
